# Massive Loss of Olfactory Receptors But Not Trace Amine-Associated Receptors in the World’s Deepest-Living Fish (*Pseudoliparis swirei*)

**DOI:** 10.3390/genes10110910

**Published:** 2019-11-08

**Authors:** Haifeng Jiang, Kang Du, Xiaoni Gan, Liandong Yang, Shunping He

**Affiliations:** 1 The Key Laboratory of Aquatic Biodiversity and Conservation of Chinese Academy of Sciences, Institute of Hydrobiology, Chinese Academy of Sciences, Wuhan 430072, China; jianghf@ihb.ac.cn (H.J.); dukang1117@foxmail.com (K.D.); ganxiaoni_ihb@163.com (X.G.); 2 University of Chinese Academy of Sciences, Beijing 100049, China; 3 Heilongjiang River Fisheries Research Institute, Chinese Academy of Fishery Sciences, Harbin 150070, China; 4 Institute of Deep Sea Science and Engineering, Chinese Academy of Sciences, Sanya 572000, China; 5 Center for Excellence in Animal Evolution and Genetics, Kunming Institute of Zoology, Chinese Academy of Sciences, Kunming 650223, China

**Keywords:** Mariana snailfish (*Pseudoliparis swirei*), olfactory receptor, hadal trench, adaptation

## Abstract

Olfactory receptor repertoires show highly dynamic evolution associated with ecological adaptations in different species. The Mariana snailfish (*Pseudoliparis swirei*) living below a depth of 6000 m in the Mariana Trench evolved degraded vision and occupies a specific feeding habitat in a dark, low-food environment. However, whether such adaptations involve adaptive changes in the chemosensory receptor repertoire is not known. Here, we conducted a comparative analysis of the olfactory receptor (OR) and trace amine-associated receptor (TAAR) gene repertoires in nine teleosts with a focus on the evolutionary divergence between the Mariana snailfish and its shallow-sea relative, Tanaka’s snailfish (*Liparis tanakae*). We found many fewer functional OR genes and a significantly higher fraction of pseudogenes in the Mariana snailfish, but the numbers of functional TAAR genes in the two species were comparable. Phylogenetic analysis showed that the expansion patterns of the gene families were shared by the two species, but that Mariana snailfish underwent massive gene losses in its OR repertoire. Despite an overall decreased size in OR subfamilies and a reduced number of TAAR subfamilies in the Mariana snailfish, expansion of certain subfamilies was observed. Selective pressure analysis indicated greatly relaxed selective strength in ORs but a slightly enhanced selective strength in TAARs of Mariana snailfish. Overall, our study reveals simplified but specific OR and TAAR repertoires in the Mariana snailfish shaped by natural selection with respect to ecological adaptations in the hadal environment. This is the first study on the chemosensation evolution in vertebrates living in the hadal zone, which could provide new insights into evolutionary adaptation to the hadal environment.

## 1. Introduction

Olfaction is a typical chemosensation that forms the sense of smell and is essential for the survival and reproduction of various organisms. Fishes evolved highly elaborate olfactory systems through which they receive, discriminate, and perceive a broad spectrum of water-soluble chemical cues that provide vital information about food location, danger avoidance, kin recognition, and spawning migration, as well as contribute to the memory of beneficial and detrimental contexts. The olfactory perception of fishes is initiated through specific interactions between odorants and olfactory receptors (ORs) expressed in sensory neurons within the olfactory epithelium; in turn, the activities of the sensory neurons are represented as distinct odorant maps in the olfactory bulb and transmitted to higher olfactory centers in the brain, where they evoke a series of physiological and behavioral responses [1].

The OR genes, which belong to the superfamily of rhodopsin-like G-protein-coupled receptors (GPCRs), form the largest multigene family in mammals. The OR gene family contains 800–1200 functional OR genes in mice, dogs, cows, and horses and more than 2000 genes in African elephants [2]. In comparison, the genomes of ray-finned fishes contain much smaller numbers of functional OR genes, ranging from 34 to 176 [3]. Each functional OR gene encodes a unique receptor protein that is tuned to a specific set of odorant molecules, and one odorant is generally recognized by diverse combinations of OR genes [4]. This combinatorial receptor coding scheme enables the olfactory system to recognize and discriminate a vast diversity of odorant information that exceeds the limited numbers of receptors. The repertoire of functional OR genes determines the spectrum of chemical signals that a species can detect and may reflect the olfactory ability of the species. Although fishes respond to various olfactory cues such as nucleotides, amino acids, amines, bile acids, steroids, and prostaglandins, few receptors from fishes were deorphanized to date, and only one, the *OR114-1* gene, was deorphanized as the olfactory receptor for the female sex pheromone prostaglandin F_2α_ [5].

Trace amine-associated receptors (TAARs), which evolved from aminergic receptors, were initially considered neurotransmitter receptors [6]. They were recognized as another main GPCR family of olfactory receptors due to the great similarity of their expression characteristics to those of ORs [7]. The TAAR gene repertoires in teleosts range from 18 in pufferfish to 112 in zebrafish and show high evolutionary dynamics with massive species-specific expansion and contraction. They detect structurally divergent amines through salt-bridge contacts at aspartates on the transmembrane α-helices III (Asp^3.32^) and/or V (Asp^5.42^) [8]. It was suggested that the dramatically expanded clade III TAARs in teleosts were derived from clade I TAARs by first gaining Asp^5.42^ and then losing Asp^3.32^, resulting in non-classical amine recognition and increasing the capacity of the teleost olfactory system to detect amines [8].

Evolution of chemosensory receptor repertoires is characterized by extensive lineage-specific expansions and deletions, and this highly dynamic process is related to the ecological requirements of the species in question [9]. Previous research showed that the number of functional and non-functional OR genes is correlated with broadly defined environmental adaptations (e.g., aquatic vs. terrestrial) [10]. Further studies documented associations between peripheral changes in OR gene families with various ecological niche adaptation contexts such as the presence of a functioning vomeronasal organ, acquisition of acute vision, dietary transitions, habitat changes, and sociality in mammalian taxa [11,12], with broad ecological adaptations in birds [13], and with adaptation to novel food resources and modifications in pheromone communication systems in insect lineages [14,15]. Moreover, adaptive shifts in sensory tuning can also result from mutations in the sequences of existing OR genes [16]. However, an association between ecological niche and OR gene family evolution is not reported in fish lineages. In addition, teleost OR repertoires show higher divergence compared to their mammalian counterparts; they include several teleost-specific OR subfamilies and substantially larger and highly species-specific TAAR repertoires [10], making them a good model for research on molecular adaptations in the olfactory system. Thus, comparative analyses of OR gene families in closely related fish species that occupy different ecological niches would shed more light on the relationship between chemosensory evolution and niche adaptation.

Hadal trenches are located at the greatest ocean depths, typically 6000–11,000 m, representing ~1–2% of the global benthic area, and they are among the most hostile environments on the planet due to their high hydrostatic pressure, darkness, limited food resources, and low temperatures [17]. However, hadal trenches host active and diverse biological communities with high apparent levels of endemism. Snailfishes (family Liparidae) are the most common hadal vertebrate species, extending deeper and reaching higher densities than other fishes and occupying habitats ranging from intertidal to depths exceeding 8100 m [18,19]. Our recent study of the morphology and genome of the Mariana snailfish *(Pseudoliparis swirei*) captured at a depth of 7034 m in the Mariana Trench revealed a series of adaptive characteristics compared to the related tide-pool-dwelling species Tanaka’s snailfish (*Liparis tanakae*) [20]. Video recordings showed that a large aggregation of Mariana snailfish individuals of different sizes were attracted to the provided bait, suggesting that olfaction plays an important role in finding food in this species [21]. Notably, as the top predator in the hadal ecosystem, the Mariana snailfish evolved a specialized feeding strategy to cope with low food availability; it possesses an inflated stomach that is typically filled with only one dominant crustacean species, *Hirondellea gigas* [20]. However, its surface relative, Tanaka’s snailfish, is a benthic fish feeder that feeds mainly on benthic fishes and consumes small amounts of benthic crustaceans, especially during its young stage [22]. The relatively simple diet of the Mariana snailfish and the limited food sources available to this species may have driven adaptive changes in its OR repertoire. In addition, the visual system of the Mariana snailfish, which is adapted to the darkness of the hadal environment, may have degenerated considerably, as evidenced by the loss of several important photoreceptor genes. Increased acuity in chemosensory abilities such as olfaction and taste as compensation for loss of vision was reported in cave fishes [23,24], whereas acceleration of OR gene loss was shown to coincide with the acquisition of acute vision in haplorhines [11]. In addition, two species of deep-sea grenadier fishes living between 2000 and 6000 m depth were reported to have undergone an ontogenetic shift in sensory orientation, with olfaction being more important than vision in large individuals [25]. Therefore, it is of great interest to examine how environmental adaptations reshape the hierarchical structure of the olfactory receptor repertoires in deep-living fishes. Here, we comparatively analyzed the evolution of the OR and TAAR gene repertoires in nine teleost fishes, with a particular focus on the evolutionary divergence between the Mariana snailfish *P. swirei* and its shallow sea relative Tanaka’s snailfish *L. tanakae*. This work provides insights into the chemosensory adaptations that took place in vertebrates living in hadal trenches.

## 2. Materials and Methods 

### 2.1. Data Source and Sequence Data Mining 

The genome sequences of the Mariana snailfish and Tanaka’s snailfish, together with those of seven other teleost fishes, were downloaded from the NCBI interface [26] or from Ensembl version 97 [27]. Of the nine genome assemblies, six were on the chromosome level, including GRCz11, GCA_000002035.4 (zebrafish), GCA_002775205.2 (platyfish), GCA_002234715.1 (medaka), FUGU5, GCA_000180615.2 (fugu), GCA_003186165.1 (turbot), and BROAD S1 (stickleback), and three were on the scaffold level (gadMor1 (cod), GCA_004335475.1 (Mariana snailfish), and GCA_006348945.1 (Tanaka’s snailfish). One Mariana snailfish individual captured at a depth of 7034 m in the Mariana Trench was sequenced using a combination of single-molecule real-time (SMRT) sequencing and paired-end sequencing. The genome assembly of the Mariana snailfish was 684 megabases (Mb) with a scaffold N50 of 418 kilobases (kb) and a completeness of 91.7% by BUSCO assessment of single-copy orthologous genes. The genome of one Tanaka’s snailfish individual collected from a depth of ~20 m was sequenced simultaneously. The genome assembly of the Tanaka’s snailfish was 534 Mb with a scaffold N50 of 1137 kb and a completeness of 89.4% [20]. The OR and TAAR sequences of seven teleosts were retrieved either from Ensembl or from published articles used as initial queries. Sequences labeled as “pseudogenes” or lacking specific subfamily names were discarded, and only the full-length sequences with the longest isoforms were selected.

The genome-wide survey was based on TBLASTN searches at the E-value cutoff of 10^−10^ using queries of ORs and TAARs. The complete coding sequences were estimated using GeneWise (ver. 2.2) [28]. These steps were conducted in a recursive fashion until no new candidates were detected in each genome. Manual inspection was conducted to obtain complete open reading frames and proper exon/intron borders. Some candidate sequences on short scaffolds were removed as duplicated if they shared more than 97% identity with another candidate sequence found on a longer mapped scaffold. Transmembrane regions were predicted using THMM2.0 [29]. The final inclusion criteria were as follows: (1) located inside the corresponding phylogenetic tree with branch support of over 80%; (2) the top BLAST hit in the non-redundant protein database resulted in confirmed ORs or TAARs; (3) sequence length between 250 and 400 amino acids (aa); (4) presence of a 7-transmembrane topology structure.

### 2.2. Phylogenetic and Orthologous Group Analysis 

We constructed a phylogenetic tree of OR genes using a total of 750 intact ORs from nine teleosts with melanocortin genes as outgroups. For TAARs, a total of 327 intact TAARs were used with lamprey aminergic receptors as outgroups. Amino-acid sequence alignments were performed using MAFFT version 7 by E-INS-I strategy with default parameters [30]. The alignments were manually edited using Jalview [31] to delete gaps with 90% tolerance. A phylogeny was constructed by the maximum-likelihood (ML) algorithm implemented in PhyML software using the SPR setting for tree improvement and χ^2^-based aLRT for branch support [32,33,34]. Trees were visualized using FigTree [35]. To facilitate gene family classification, we did not attempt to annotate all the identified ORs and TAARs but only updated the zebrafish ORs and TAARs identified in the present study based on previously annotated names [7,36]. Genes were named according to the clustering relations of annotated family/subfamily and numbered sequentially according to genomic position, if known.

### 2.3. Estimation of Gene Gain and Loss Events

To estimate the gene gain and loss events of ORs and TAARs during the evolution of these teleost species, the tree reconciliation method implemented in NOTUNG 2.9 [37] was conducted. The species tree topology was taken from previous studies [20,38], while the gene tree topology was taken from our ML tree. Gene gains and losses were inferred by reconciliation of the gene tree with the species tree, producing resolved incongruence topologies (collapsing branches with branch supports lower than 0.95) by the parsimony principle to avoid overestimation of the gene turnover and the estimated minimum number of gene gain and loss events.

### 2.4. Chromosomal Distribution and Gene Duplication

The identified ORs and TAARs of the Mariana snailfish and Tanaka’s snailfish were mapped to stickleback chromosomes using MCScanX [39]; the minimum block size for collinear pairs was 25 orthologous pairs. Collinear analysis maps were constructed using TBtools [40] to display the collinear relationship of ORs and TAARs obtained from the three species. To better explore the duplication patterns of ORs and TAARs in the two closely related species, we conducted a collinear analysis using MCScanX with default parameters. Prior to MCScanX analysis, reciprocal BLAST and self-BLAST with E-value < 1 × 10^−5^ were conducted to call the homology of the OR and TAAR genomic regions in the two genomes. Genes were classified as singleton, dispersed (may have arisen from transposition), proximal (may have arisen from small-scale transposition or from tandem duplication and insertion), tandem duplication, whole-genome duplication (WGD), or segmental duplication.

### 2.5. Selection Analysis

To investigate the selective pressures that shaped the evolution of the Mariana snailfish OR gene family, we classified orthologous groups of genes from the Mariana snailfish and Tanaka’s snailfish based on our phylogenetic analysis. In addition, we conducted comparative genomic analysis using OrthoVenn2 [41] to identify the orthologous clusters. Based on our phylogenetic tree and on the annotation of orthologous clusters, we defined monophyletic orthologous groups for each OR and TAAR subfamily shared by the Mariana snailfish and Tanaka’s snailfish that consisted of at least four ortholog copies including paralogs. To identify genes that underwent positive selection, we used the adaptive branch-site relative effects likelihood (aBSREL) method implemented in the Datamonkey web server [42] to detect branches with *dN/dS* ratios significantly >1, indicating positive selection along these branches [43]. To test whether purifying selection in the Mariana snailfish was relaxed, we applied RELAX implemented in the Datamonkey web server, using genes of the Mariana snailfish as test branches. RELAX detects selective strength based on a codon-based, branch-site random-effects method in which a selection intensity parameter *k* is used to modulate the divergence degree of the nonsynonymous/synonymous substitution rate ratio (ω) [44]. Values of *k* < 1 represent selective relaxation, whereas values of *k* > 1 represent selective intensification; the significance of the results is tested by comparing alternative models to the null model (*k* = 1) using the likelihood-ratio test.

In addition, we calculated the *dN/dS* ratios for the orthologous groups that showed signatures of positive selection using single-likelihood ancestor counting (SLAC) [45], as well as the fixed-effects likelihood (FEL) method [46]. The probability cutoff *p* was set at <0.1, and only positions that showed consensus results by both methods were considered.

## 3. Results

### 3.1. Substantially Reduced Number of OR Genes in the Mariana Snailfish Caused by Pseudogenization

**Figure 1:**
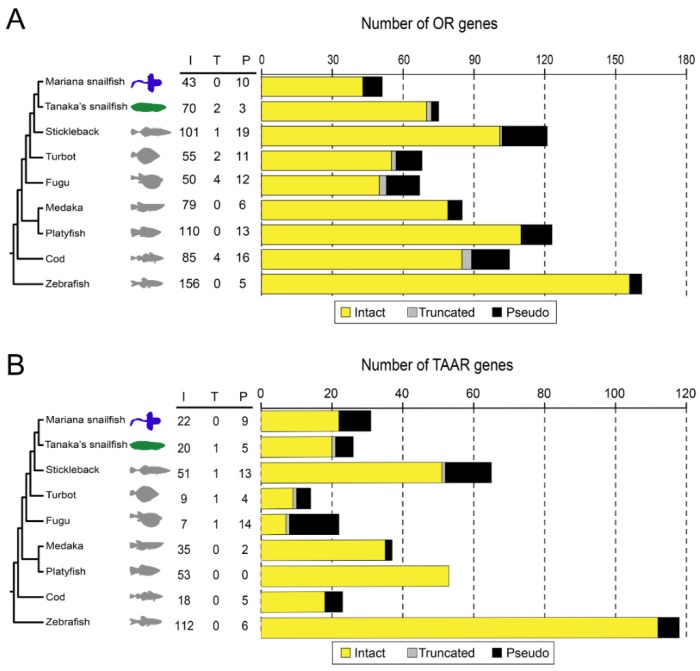
Numbers of olfactory receptor (OR) genes (**A**) and trace amine-associated receptor (TAAR) genes (**B**) in the genome sequences of nine fish species. “I”, “T”, and “P” represent the number of intact genes, truncated genes, and pseudogenes, respectively. The phylogeny of nine fish species was taken from previous studies.

We retrieved the complete OR and TAAR gene repertoires of nine teleost fishes (Mariana snailfish, Tanaka’s snailfish, turbot, fugu, medaka, platyfish, cod, and zebrafish) using a recursive search strategy (Figure 1). The identified ORs and TAARs were classified into three categories depending on their potential for functionality: putative functional (intact) genes, truncated genes, and pseudogenes. A functional gene is an intact gene that has initiation and stop codons at the proper positions and without nonsense or frameshift mutations in the coding sequence. A truncated gene is defined as a partial sequence of an intact gene and may be reassigned as a functional gene or a pseudogene if the quality of the genome sequence data is improved. However, it should be noted that putative functional genes may actually be pseudogenes since deleterious mutations in a promoter region can also prevent the expression of an intact gene. Therefore, our enumeration of pseudogenes in this study is conservative. The numbers of OR and TAAR genes identified in the present study are similar to the numbers previously reported (Table S1, Supplementary Materials), suggesting that the genes identified by our pipeline are reliable. Moreover, among the two snailfish genomes, we only identified few truncated OR and TAAR genes in the Tanaka’s snailfish, confirming the completeness of OR and TAAR repertoires. All sequences of the identified OR and TAAR genes are provided in Supplementary Data 1.

Gene repertoire size varied substantially among teleost lineages, ranging from 53 OR genes in the Mariana snailfish to 161 OR genes in the zebrafish (Figure 1A). Notably, the number of OR genes in the Mariana snailfish (53) was much lower than that in Tanaka’s snailfish (75), while the numbers of TAAR genes (31 in the Mariana snailfish, 26 in Tanaka’s snailfish) were comparable (Figure 1B), highlighting the difference in ORs between the two species. This result was more obvious when only the functional ORs and TAARs (43 vs. 70 functional ORs and 22 vs. 20 functional TAARs in the two species) were considered. Notably, the percentage of OR pseudogenes in the Mariana snailfish (23.3%) was significantly larger than that in Tanaka’s snailfish (4%); among the pseudogenes, frameshift mutations were more frequent than nonsense mutations. Thus, the contraction in OR family size in the Mariana snailfish was probably the result of gene deletion caused by pseudogenization.

### 3.2. An Overall Decreased Size of OR Gene Families and a Reduced Number of TAAR Gene Families in the Mariana Snailfish Compared to Tanaka’s Snailfish

**Figure 2:**
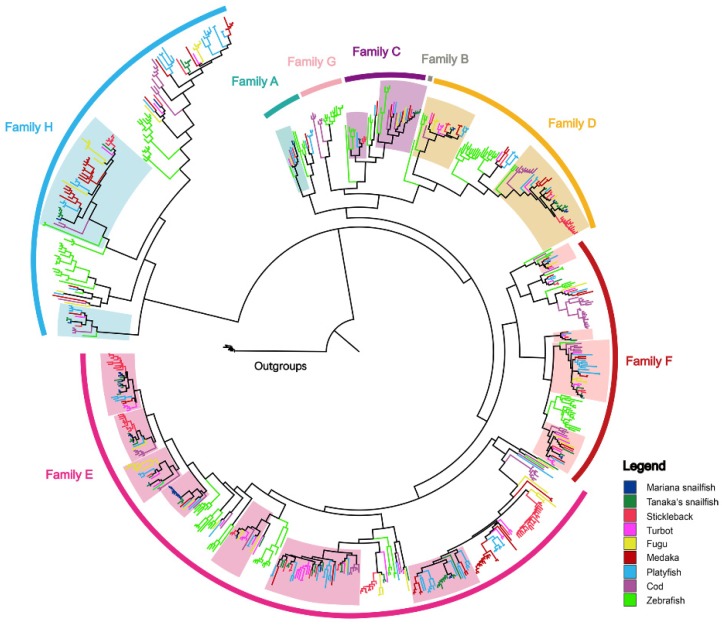
A phylogenetic tree of ORs from nine fish species (legends are indicated on the right side of the figure). Melanocortins from seven teleosts were used as an out-group. The orthologous groups containing the Mariana snailfish and Tanaka’s snailfish ORs are indicated by shading.

**Figure 3:**
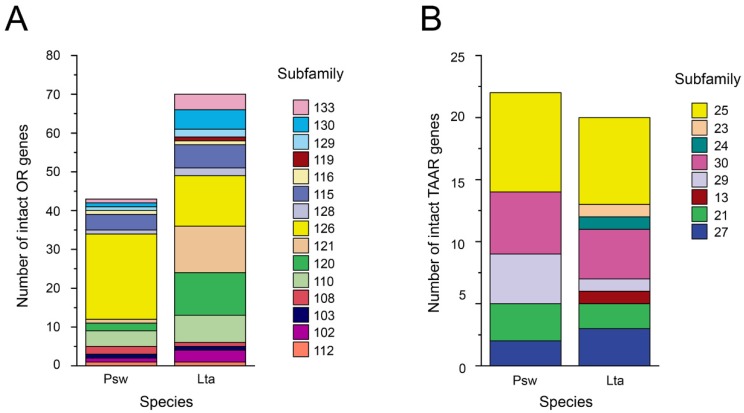
Comparison of OR (**A**) gene number and TAAR (**B**) gene number. The number of intact ORs and TAARs between the Mariana snailfish and Tanaka’s snailfish is shown by a stacked bar graph. Each of the subfamilies is indicated by a different color. Abbreviation of species names is as follows: Psw, Mariana snailfish; Lta, Tanaka’s snailfish.

Phylogenetic analysis of the ORs in the Mariana snailfish and Tanaka’s snailfish, together with the seven other fish species, identified seven OR families, family A to family H (Figure 2 and Figure S1, Supplementary Materials). Families B and G were not found in either the Mariana snailfish or Tanaka’s snailfish. The identified ORs in these two closely related species were organized into 15 subfamilies (Figure 3A). Of these subfamilies, 10 had fewer members in the Mariana snailfish than in Tanaka’s snailfish, especially subfamily 120 and subfamily 121, which had two and one members, respectively, in the Mariana snailfish and 11 and 12 members, respectively, in its shallow-living counterpart. Interestingly, the number of OR genes in the most expanded subfamily, subfamily 126, in the Mariana snailfish (22) was much greater than that in Tanaka’s snailfish (13), suggesting that subfamily 126 may play a central role in olfactory detection in the Mariana snailfish. According to the family/subfamily classification of OR genes (Figure 2 and Figure 3A), the OR subfamilies represented in the repertoires of the two closely related species showed complete overlap, indicating that the gene duplication events that gave rise to the major OR families occurred prior to the divergence of these two species. However, the fact that fewer OR genes were found in the OR families of the Mariana snailfish than in the OR families of Tanaka’s snailfish suggests that independent gene losses occurred in the Mariana snailfish OR repertoire. Given that the presence of a greater number of paralogs in the same subfamily suggests that the organism may possess a greater number of olfactory sensory neurons that bind a specific odorant, this overall contraction in OR gene families may indicate decreased sensitivity in the detection of the corresponding chemical cues, except in the case of subfamily 126.

**Figure 4:**
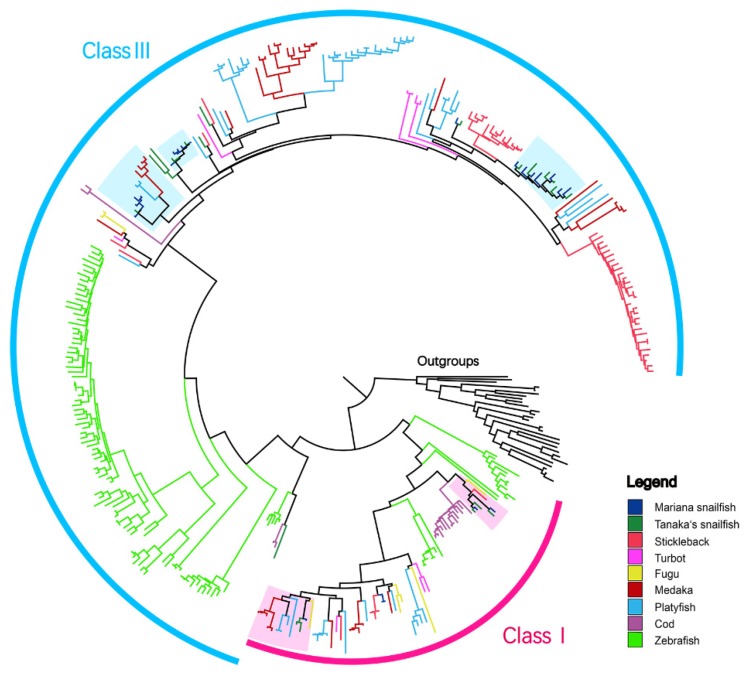
A phylogenetic tree of TAARs from nine fish species (legends are indicated on the right side of the figure). Lamprey aminergic receptors were used as an out-group. The orthologous groups containing both of the Mariana snailfish and Tanaka’s snailfish TAARs are indicated by shading.

A phylogenetic tree of TAARs is displayed in Figure 4. The phylogenetic analysis shows that the teleost TAAR gene family can be represented by two main clades, Class I and Class III. We found no subfamilies that consisted of simple 1:1 orthologous genes in the Mariana snailfish and Tanaka’s snailfish; the TAARs in the expanded subfamilies for the two species nested together in the phylogenetic tree (Figure S2, Supplementary Materials), suggesting a highly conserved evolution of the TAAR family in the two species. The phylogenetic tree also identified snailfish-specific expansions (TAAR30) shared by the two snailfish lineages, suggesting potential functional specialization. The Class I subfamilies of the Mariana snailfish and Tanaka’s snailfish were TAAR21 and TAAR27 (Figure 3B). However, there were fewer Class III subfamilies in the Mariana snailfish (TAAR29, 30 and 25) than in Tanaka’s snailfish (TAAR13, 23, 24, 25, 29, 30). The presence of fewer subfamilies in the Mariana snailfish may indicate that this species has a decreased capability to detect a broad range of amines. Notably, the numbers of TAARs in the individual subfamilies were comparable in the two species except in the case of TAAR subfamily 29, which had four members in the Mariana snailfish and only one in Tanaka’s snailfish, indicating a slightly increased sensitivity of detection of particular amines.

### 3.3. Highly Dynamic Gene Birth and Death Shaped the OR and TAAR Evolution

**Figure 5:**
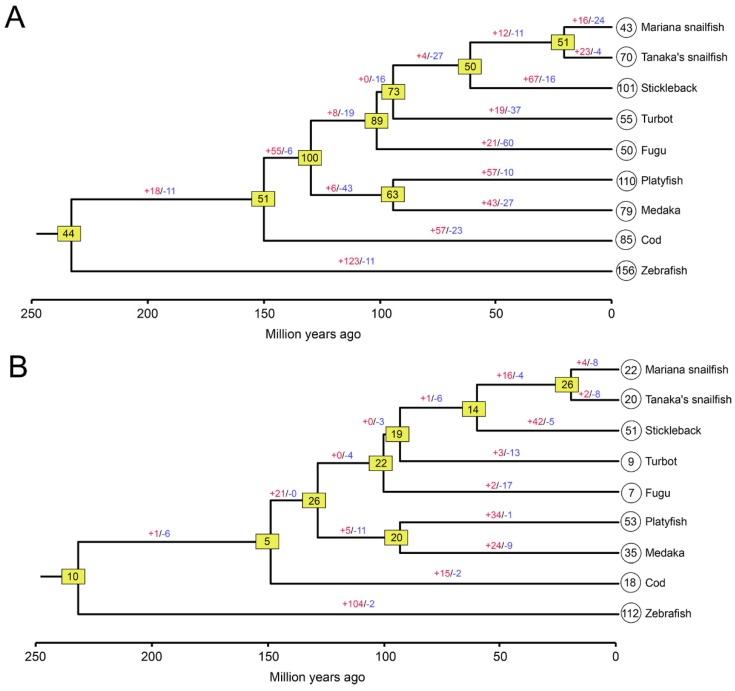
Estimation of gene gain and loss events and number of ORs (**A**) and TAARs (**B**) in the ancestral species. Number of gene gains and losses are shown on each branches with + and −, respectively. Numbers in yellow boxes indicate the number of ancestral nodes, and the numbers of intact genes in extant fish species are shown in circles. The approximate divergence times of the nine fish species was inferred from references.

To further characterize the evolutionary dynamics of OR and TAAR gene repertoire size in the teleost lineage, we estimated the number of gene gain and loss events and the ancestral gene (Figure 5A). In accordance with the birth-and-death model, our analysis showed that highly dynamic changes in the number of ORs occurred during the evolution of teleosts. The greatest increase in the number of genes was observed in zebrafish (123), and the highest gene losses were detected in fugu (60). The common ancestor of the Mariana snailfish and Tanaka’s snailfish, which lived approximately 20 million years (MY) ago, was estimated to have 51 OR genes, and various gene gains and losses appear to have occurred after the divergence of the two species. Gene gains in the Mariana snailfish (16) were fewer than those in Tanaka’s snailfish (23), but substantially more gene losses occurred in the Mariana snailfish (24) than in Tanaka’s snailfish (4), leading to a greatly contracted OR gene repertoire in the Mariana snailfish. Our findings suggest a rapid rate of receptor evolution meditated by a birth-and-death process that can fix variations over relatively short spans of time.

In the TAAR gene family, the recent lineage-specific expansions and contractions resulted in a lower number of estimated ancestral genes than were found in the OR gene family (Figure 5B). For example, the number of common ancestors of TAARs in the nine fish species was estimated to be 10, far fewer than the 44 estimated common ancestors of ORs. The highest gene gains (104) and losses (17) were detected in zebrafish and fugu, respectively. In the Mariana snailfish and Tanaka’s snailfish, the number of ancestral TAARs was estimated to be 26, greater than the current number of TAARs in the two species. However, the gene gains in the Mariana snailfish (four) were greater than those in Tanaka’s snailfish (two), while the gene losses in the two species were the same (eight), leading to the presence of similar numbers of TAARs in the two lineages. The similar gene turnover and repertoire size of the TAAR gene families in the Mariana snailfish and Tanaka’s snailfish revealed a comparable reliance on the function of the TAAR gene family, which is ecologically relevant and co-varies in size in the two closely related species.

### 3.4. Genomic Organization Reveals More Orthologous Regions in ORs than in TAARs

In the six chromosome-level assemblies (zebrafish, platyfish, medaka, fugu, turbot, and stickleback), we found that the identified teleost ORs were clustered in large tandem arrays and that most of them were scattered on four chromosomes; one exception was the fugu, in which most ORs were scattered in small scaffolds and only a few OR clusters were found on three chromosomes. The number of ORs on the four chromosomes ranged from four to 67; one chromosome often had many fewer ORs than the other three chromosomes, and, in a few cases, OR genes existed as solitary genes (Supplementary Data 2). The TAARs identified in the present study were found to be present in tandem arrays on one to three chromosomes and, in a few cases, as solitary genes. This tandem organization of ORs and TAARs is consistent with genesis of the clusters by recurrent local gene duplication. Within the clusters, closely related genes resided together in accordance with their phylogenetic relationship, reflecting likely evolutionary proximity (Figures S1 and S2, Supplementary Materials). Moreover, genes within the same subfamily usually shared the same transcriptional orientation, suggesting tandem duplication as a mechanism of expansion within a subfamily.

The OR genes found in the Mariana snailfish and Tanaka’s snailfish were distributed on 17 and 12 scaffolds, respectively, with the largest clusters harboring 15 (scaffold 53) and 16 (scaffold 34) OR genes, respectively. The number of isolated OR genes in single scaffolds accounted for 13.2% of the Mariana snailfish OR repertoire and for 5.3% of the Tanaka’s snailfish OR repertoire. Four of the Mariana snailfish scaffolds and four of the Tanaka’s snailfish scaffolds containing OR genes could be mapped onto three stickleback chromosomes containing larger OR clusters (Figure S3, Supplementary Materials). Therefore, the scattered scaffolds containing ORs in the current genome could be anchored to tighter clusters with greater scaffold lengths and completeness. Furthermore, the positions of the eight orthologous pairs identified in the Mariana snailfish and the stickleback were the same as those for Tanaka’s snailfish, revealing the presence of highly conserved syntenic blocks, a factor that may be important in evolution. The TAAR genes found in the Mariana snailfish and in Tanaka’s snailfish were distributed on seven scaffolds in each species, with the largest clusters harboring 20 (scaffold 14) and 15 (scaffold 2831) TAAR genes, respectively. However, we found only one orthologous pair of TAAR genes in the stickleback and the Mariana snailfish.

### 3.5. Collinear Analysis Reveals Orthologous Pairs Formed Prior to the Divergence of the Mariana Snailfish and Tanaka’s Snailfish

**Figure 6:**
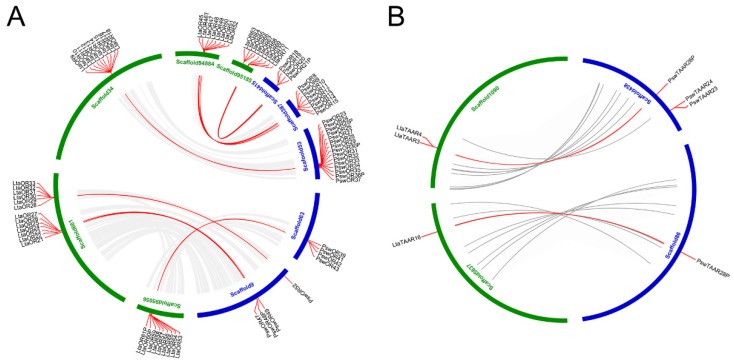
Identification of orthologous blocks between the Mariana snailfish and Tanaka’s snailfish. (**A**) Six pairs of OR orthologous blocks are anchored by 13 pairs of OR genes linked with orange lines. **(B**) Two pairs of TAAR orthologous blocks are anchored by two pairs of TAAR genes linked with orange lines. Scaffolds from Mariana snailfish and Tanaka’s snailfish are indicated by blue and green, respectively. Tandem duplicated genes are indicated with orange lines along its own scaffold.

Collinear analysis was conducted to characterize the duplication patterns and the conservation of ORs and TAARs between the Mariana snailfish and Tanaka’s snailfish. Thirteen orthologous OR gene pairs were distributed on six pairs of orthologous blocks in the two species (Figure 6A, Supplementary Data 3). Tandem duplications accounted for 62.3% of the OR genes in the Mariana snailfish and 73.0% of the OR genes in Tanaka’s snailfish, together with a small percentage of transposition events. In contrast, we observed only two orthologous TAAR pairs belonging to Class I and Class III in the two species (Figure 6B, Supplementary Data 3). Tandem duplications were the main way in which TAAR genes arose, accounting for 68.8% of the TAAR genes in the Mariana snailfish and 80.8% of the TAAR genes in Tanaka’s snailfish. Considering that collinearity could have been introduced after the divergence, the collinear pairs of OR and TAAR genes found in this study might have originated prior to the divergence of the two species, while the other members of these gene families may have been derived from more recent tandem duplication events.

### 3.6. Strong Relaxed Selective Strength in ORs But Slightly Enhanced Selective Strength in TAARs of the Mariana Snailfish

To better understand the evolutionary constraints on ORs and TAARs in the Mariana snailfish and in Tanaka’s snailfish, positive selection analysis and relaxed selection analysis were conducted. We defined 18 orthologous groups that included both the Mariana snailfish and Tanaka’s snailfish (Figure S1, Supplementary Data 4, Supplementary Materials). In the Mariana snailfish, aBSREL found only one OR gene in orthologous group OGn18, exhibiting a signature of positive selection (Figure S1, Supplementary Data 5, Supplementary Materials). In contrast, in Tanaka’s snailfish, six OR genes organized into five orthologous groups (OGn3, OGn11, OGn13, OGn15, and OGn17) showed evidence of positive selection. Furthermore, we ran RELAX to test whether the strength of natural selection was relaxed in ORs of the Mariana snailfish. As expected, the OR genes in six orthologous groups of the Mariana snailfish showed significantly relaxed selection strength, while no intensified selection was detected (Table 1). These results reveal an apparently relaxed selection in the OR gene family of the Mariana snailfish.

In contrast to the ORs, aBSREL found two TAARs in the Mariana snailfish in two of five orthologous groups, while none of the TAARs in Tanaka’s snailfish exhibited evidence of positive selection (Figure S2, Supplementary Data 5, Supplementary Materials). RELAX found no Mariana snailfish TAARs that showed relaxed selection, but significantly intensified selection was detected in one orthologous group (OGn1) (Table 1). These results suggest a slightly enhanced selective strength on the TAAR gene family of the Mariana snailfish. Further calculation of *dN/dS* ratios in the TAAR OGn1 by SLAC and FEL revealed two sites respectively located at the N-terminus and in extracellular loop III, showing positive selection (Figure S4, Supplementary Data 5, Supplementary Materials), inconsistent with previous reports that ligand-binding sites are commonly located within transmembrane regions [6,7].

## 4. Discussion

The complexity of chemosensory receptor repertoires was suggested to reflect species-specific ecological needs [47]. As the world’s deepest-living fish, the Mariana snailfish, which is adapted to a hadal environment, might have evolved degenerated olfaction due to its specific dietary habits but also enhanced olfaction to compensate for its deteriorated vision. Here, based on a comparative analysis of the OR and TAAR repertoires of the Mariana snailfish with those of its shallow-sea relatives, we report that simplified but specific OR and TAAR repertoires in the Mariana snailfish are consistent with the changes in selective constraints. The distinct evolutionary patterns strongly support the idea that birth-and-death and relaxed selection resized and reshaped the OR and TAAR gene families [48].

The teleost OR and TAAR gene families evolved under the birth-and-death mode of evolution, as evidenced by the occurrence of repeated gene duplication, pseudogenization, and loss. Pseudogenization, as the first step in gene loss, is a major contributor to decreases in repertoire size. For instance, two-thirds of the OR repertoire of primates such as humans and chimpanzees is represented by pseudogenes; pseudogenes also represent six-sevenths of the OR genes in an avian species, the chicken [10]. The OR gene repertoires of the secondary-adapted cetacean lineage (the minke whale *Balaenoptera acutorostrata*, the dwarf sperm whale *Kogia sima*, Dall’s porpoise *Phocoenoides dalli*, and Steller’s sea lion *Eumetopias jubatus*) lost large numbers of OR genes accompanied by pseudogenization [49]. Considering that the mutation rate across the entire genome of the Mariana snailfish is lower than that in Tanaka’s snailfish [20], the substantially higher percentage of pseudogenes in its OR repertoire indicates that the Mariana snailfish lost more genes than Tanaka’s snailfish following their divergence.

It was suggested that the number of functional OR genes is correlated with specific environmental adaptations [11]. Thus, the massive loss in functional ORs and the retention of a similar number of functional TAARs in the Mariana snailfish compared to its shallow-living counterpart suggests that the specific feeding habitat of the Mariana snailfish resulted in decreased dependence on OR gene function, while its reliance on TAARs may still be important. The degeneration of OR gene repertories in the Mariana snailfish can be explained by the fact that hadal trenches have much less abundance and biomass of organisms due to many constrained factors, such as extremely high hydrostatic pressure, low temperatures, absence of solar light, and limited food sources [17], resulting in a relatively simple chemical environment; thus, many olfactory cues that crucial for their survival are absent or become less important for this top predator in the hadal ecosystem. In contrast, organic debris, such as the carcasses of shallower-living fauna falling from upper layers, is the main food source for species in the hadal zone [50]; during its microbial decomposition, this debris releases a variety of amines that attract scavenging fauna, which can be detected by the TAARs expressed in olfactory neurons. Therefore, the function of TAAR genes to detect amines is critical for the Mariana snailfish. However, the observation of the bait-attending species suggested that the Mariana snailfish is exclusively predatory; it does not feed directly on the bait but rather suction feeds on swarms of scavenging amphipods that are attracted to the bait [21]. In fact, the comparable size of the functional TAAR repertoire in the two closely related species does not indicate enhanced amine detection in the Mariana snailfish, consistent with the fact that the Mariana snailfish did not experience a feeding shift to scavenging during its adaptation to the hadal environment after divergence from its shallow-sea relatives.

ORs and TAARs are the main duplicated gene families, and the lineage-specific expansion of certain subfamilies probably led to an increase in sensitivity to particular chemical cues that may have been adaptive in certain chemical contexts. Generally, it is likely that the number of OR subfamilies correlates with odorant discrimination ability because ORs that share less sequence homology are expected to bind different sets of odorants, while the number of ORs in certain subfamilies is correlated with sensitivity to particular odorants due to the presence of similar binding sites that interact with similar molecular structures [12,51,52]. Consistent with this possibility, we found that distinct subfamilies of both the OR and TAAR gene families evolved independently; this can be considered a response to different chemical cues that are relevant in each species’ particular ecological niche. Accordingly, the contracted or lost OR and TAAR gene families of the Mariana snailfish may reflect a decreased capability to detect olfactory cues that are absent from its environment, but the presence of expanded gene families appears to increase the Mariana snailfish’s sensitivity to particular olfactory cues that are common or critical in its chemical environment. Overall, the comparative analysis of OR and TAAR evolution in the two closely related species reveals a simplified but specialized OR and TAAR gene repertoire in the Mariana snailfish that may be related to the simple chemical ecology of the hadal environment. However, information on the molecules that are recognized by individual receptors and how snailfish respond to these odorants is necessary to further elucidate this process.

Previous studies showed that the majority of OR genes are organized into clusters and that isolated OR genes rarely occur in vertebrate genomes [53]. In mammalian genomes, ORs are widely distributed; for instance, OR gene clusters reside on 16 and 21 chromosomes in pigs [54] and humans [55], respectively. We found that the ORs in teleosts are clustered in tandem arrays and distributed on four chromosomes and that orthologous ORs of the Mariana snailfish and Tanaka’s snailfish are located in chromosomal regions homologous to those in the stickleback, revealing a high degree of conservation of the genomic landscape. Most of the teleost TAARs in the present study were distributed in a way that is consistent with previous findings that TAARs occur in tandem arrays on one to three chromosomes and as a few solitary genes [7,56,57], unlike TAARs in tetrapods, which are all found in a single cluster in the genome [56]. In contrast, the collinear analysis revealed a less conserved genomic location of TAARs that may be attributed to the birth-and-death process in different lineages, as an ancestral gene may expand into a large subfamily in one lineage but become pseudogenized or deleted in another. The different degrees of conservation of the genomic architectures of the OR and TAAR gene families suggest that genomic rearrangements have a relatively weak influence on the highly dynamic evolutionary history of these families relative to local molecular mechanisms. A similar observation was also reported in insect lineages [58].

We found no WGD in the evolution of ORs and TAARs in the two species and confirmed that tandem duplication was the major driving force for the expansion of the OR and TAAR gene families [36]. In fact, tandem duplication that gives rise to a clustered organization of ORs and TAARs plays an important role in gene regulation. For instance, expression of a single OR coincides with the nuclear convergence of OR gene clusters, which promotes interchromosomal interactions and enhances OR gene choice. This interaction results in the expression of ORs in mature olfactory sensory neurons (mOSN) in a monogenic, monoallelic, and stochastic fashion in which each neuron expresses only one OR gene [1].

Relaxed selection can occur in various contexts such as the removal of a functional constraint (e.g., a new ecological niche) or a reduction in selection efficiency [44]. Relaxation of selective constraints after gene duplication can facilitate neofunctionalization and subfunctionalization, permitting conserved chemical communication or local adaptation. An important side effect of relaxed selection pressure is increasing pseudogenization leading to gene loss [48]. Therefore, the contraction of OR subfamilies and higher pseudogenization in the Mariana snailfish compared to its shallow-living relatives may have been due to relaxed selection resulting from the removal of functional constraints, i.e., a lack of olfactory-dependent natural selective pressure in the hadal zone, where natural habitats lack food diversity. In addition, the interaction between olfactory cues and receptors of the olfactory epithelium is also weakened under high hydrostatic pressure, while lower temperature slows the diffusion of odorant molecules [59], and higher pollution in the hadal zone [60] hampers the normal function of olfactory detection. Nevertheless, the massive loss of ORs in the Mariana snailfish does not necessarily indicate a decrease in olfactory ability, which includes both sensitivity to odorants and discrimination of odorants. More evidence from behavioral and neuroanatomical studies of the peripheral sense organs is necessary to elucidate the connection between olfactory receptor gene repertoire and olfactory ability.

## 5. Conclusions

This study reveals the first comparative analysis of the chemosensation evolution in the Mariana snailfish, the world deepest-living vertebrate. We found that the Mariana snailfish has many fewer ORs and a much higher ratio of OR pseudogenes compared to Tanaka’s snailfish but that it retains similar numbers of TAARs. The massive loss of ORs but not TAARs in the Mariana snailfish reflects its different reliance on olfactory function. Furthermore, distinct gene families/subfamilies evolved independently, resulting in simplified but specialized OR and TAAR gene repertoires. This dynamic tuning correlates with the adaptation of the species to the simple chemical ecology of the hadal environment. The overall contraction of the OR gene family also coincides with strong signatures of relaxed selective pressure. Our findings provide new evidence that birth-and-death and relaxed selection are the dominant processes that resize and reshape the OR and TAAR repertoires. Finally, we consider that comparative analysis of closely related species living in different ecological systems can elucidate chemosensory adaptations at a finer scale, thus providing important insights into the evolutionary ecology of chemosensation.

## Figures and Tables

**Table 1:** Test for shift in selective pressures using RELAX. OR—olfactory receptor; TAAR—trace amine-associated receptor.

Gene Family	Gene Subfamily	Orthologous Groups	Selection Intensity *k*	*p*-Value
OR				
Family A	OR112	OGn3	0.58	<0.001 *
Family C	OR102	OGn5	0.10	0.568
	OR103	OGn4	1.63	0.500
Family D	OR108	OGn6	0.46	0.456
	OR110	OGn7	1.37	0.084
Family E	OR120	OGn12	0	0.134
	OR121	OGn13	0.51	0.042 *
	OR126	OGn15	0.80	0.283
		OGn16	1.00	1.000
		OGn17	1.25	0.415
		OGn18	0.19	1.000
	OR127	OGn14	0.62	0.260
Family F	OR115	OGn9	0.22	0.592
		OGn10	0.11	0.013 *
		OGn11	2.17	0.108
	OR116	OGn8	0.31	0.007 *
Family H	OR129	OGn1	0	0.049 *
	OR130	OGn2	0	0.009 *
TAAR				
Class I	21	OGn2	8.05	0.102
	27	OGn1	4.57	0.020 *
Class III	29	OGn3	0.73	0.076
	30	OGn4	8.24	0.177
	25	OGn5	0.82	0.266

* Indicates a significant RELAX test.

## Supplementary Materials

The following are available online at https://www.mdpi.com/2073-4425/10/11/910/s1: Figure S1: Phylogenetic tree of ORs from nine fish species with names. Eighteen orthologous groups containing ORs from both the Mariana snailfish and Tanaka’s snailfish are organized in eight families. Bootstrap supports are shown. Stars indicate ORs of the Mariana snailfish or Tanaka’s snailfish under positive selection, Figure S2: Phylogenetic tree of TAARs from nine fish species with names. Five orthologous groups containing ORs from both Mariana snailfish and Tanaka’s snailfish are organized in 25 families. Bootstrap supports are shown. Stars indicate TAARs of the Mariana snailfish or Tanaka’s snailfish under positive selection, Figure S3: Synteny analysis of OR and TAAR genes between the Mariana snailfish and Tanaka’s snailfish with stickleback. Gray lines in the background indicate the collinear blocks, while the red lines highlight the syntenic OR gene pairs, and purple lines highlight the syntenic TAAR gene pairs, Figure S4: Positive selection in orthologous group OGn1 of TAAR. Predicted selective pressure for individual codons is shown as consensus *dN/dS* values of FEL and SLAC (negative selection in blue, positive selection in red). The schematic representations were drawn based on consensus TM predictions for the codon-based nucleotide alignment; Table S1: Comparison of functional OR and TAAR gene counts between our study and previous reports; Supplementary data 1: Amino-acid and nucleotide sequences of all ORs and TAARs identified in this study, Supplementary data 2: The genomic location of all ORs and TAARs identified in this study, Supplementary data 3: The orthologous blocks and duplication types identified in the Mariana snailfish and Tanaka’s snailfish, Supplementary data 4: Orthologous groups of ORs and TAARs identified by OrthoVenn2, Supplementary data 5: The detailed results of aBSREL and *dN/dS* analysis.
