# Supplementary material for: Massive Loss of Olfactory Receptors But Not Trace Amine-Associated Receptors in the World’s Deepest-Living Fish (Pseudoliparis swirei)
Source: Genes (Basel). 2019 Nov 8;10(11):910. doi: 10.3390/genes10110910 (PMC6895882; doi:10.3390/genes10110910)
Supplement: Supplementary file 1 [file genes-10-00910-s001.zip › Table S1 .pdf]

Supplementary Table 1 Comparison of functional OR and TAAR gene counts between our study with previous reports.

Species	Number of ORs (This study)	Reported ORs (Niimura's study)[a]	Number of TAARs (This study)	Reported TAARs (Hussain' study)[b]
Zebrafish	156	154	112	112
Stickleback	101	102	51	48
Medaka	79	68	35	25
Fugu	50	47	7	18

[a] Y. Niimura, Olfactory Receptor Multigene Family in Vertebrates: From the Viewpoint of Evolutionary Genomics, Curr Genomics, 13 (2012) 103-114.

[b] A. Hussain, L.R. Saraiva, S.I. Korsching, Positive Darwinian selection and the birth of an olfactory receptor clade in teleosts, Proc Natl Acad Sci U S A, 106 (2009) 4313-4318.
