# Supplementary figures and images for: Massive Loss of Olfactory Receptors But Not Trace Amine-Associated Receptors in the World’s Deepest-Living Fish (Pseudoliparis swirei)
Source: Genes (Basel). 2019 Nov 8;10(11):910. doi: 10.3390/genes10110910 (PMC6895882; doi:10.3390/genes10110910)

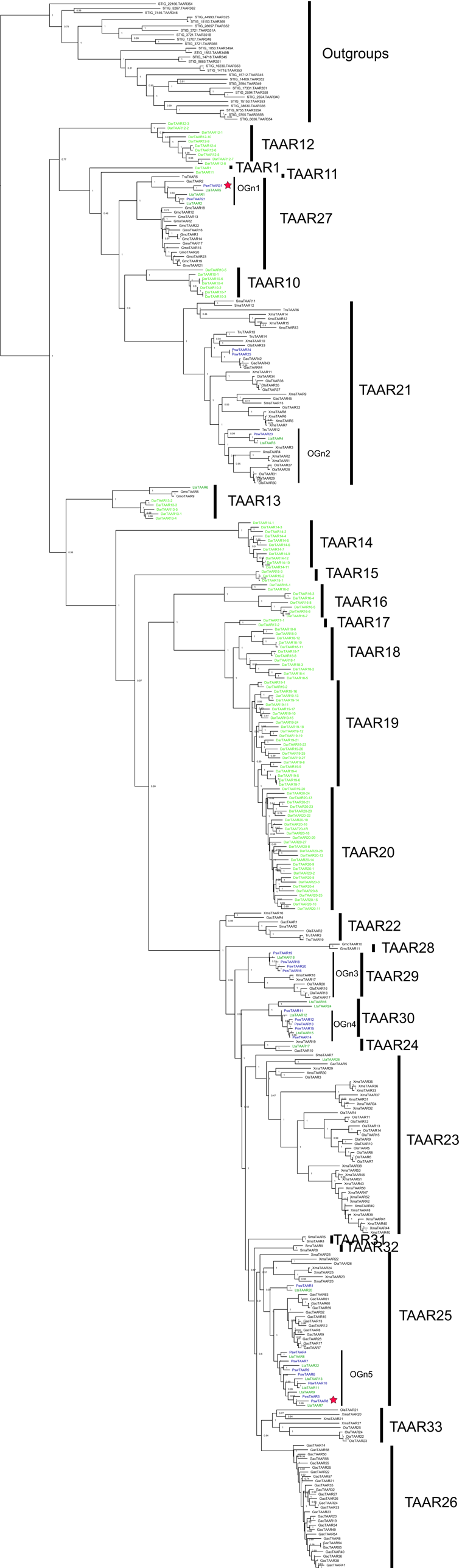

Supplement: Supplementary file 1 [file genes-10-00910-s001.zip › Figure S2.pdf]

**Mariana snailfish**

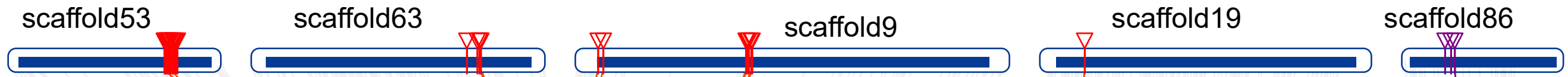

**Stickleback**

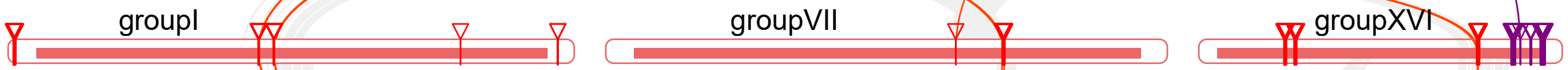

**Tanaka's snailfish**

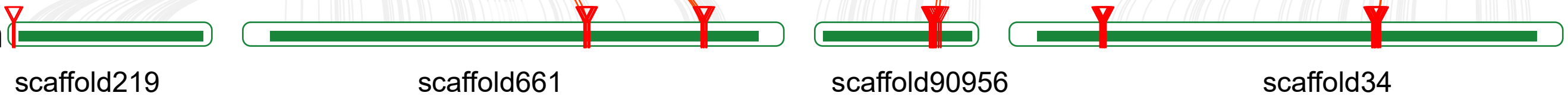

Supplement: Supplementary file 1 [file genes-10-00910-s001.zip › Figure S3.pdf]

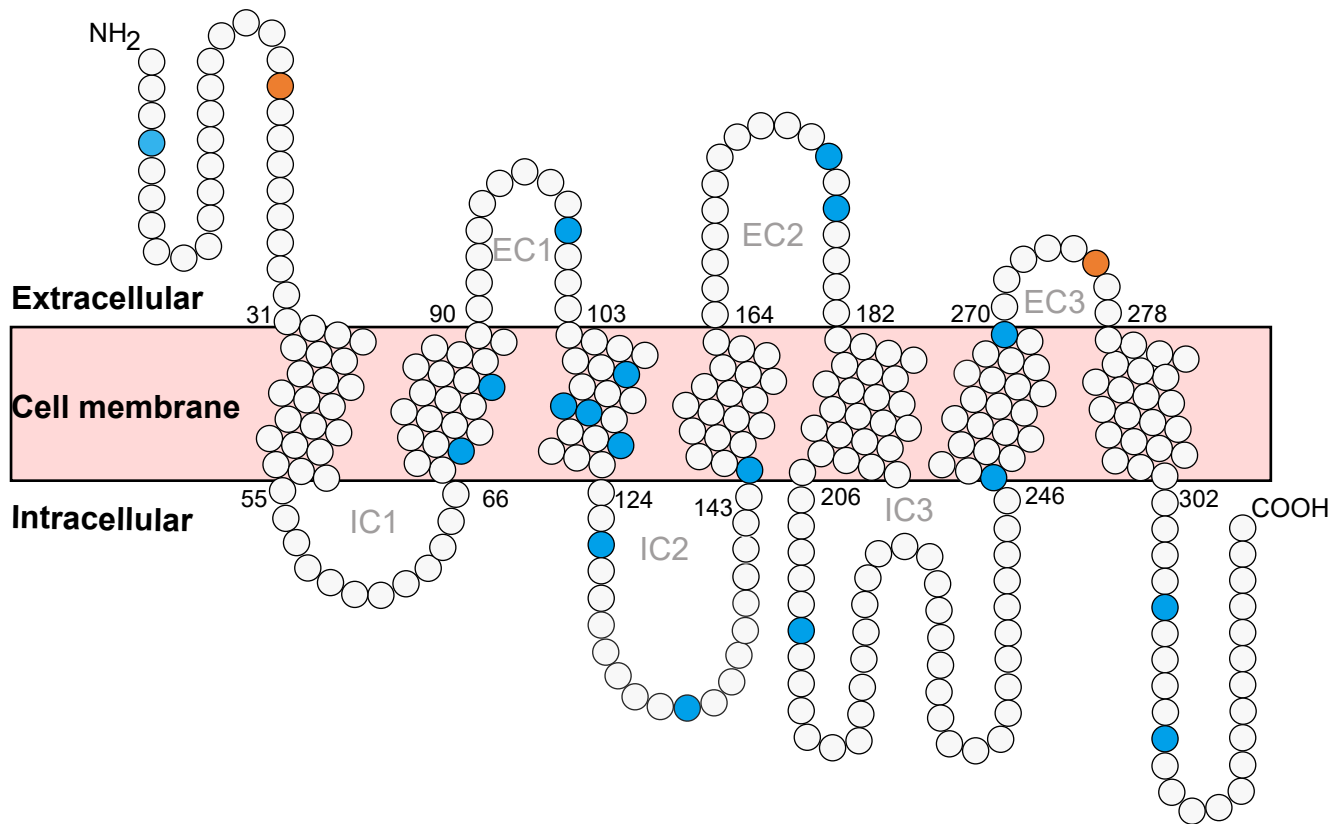

Supplement: Supplementary file 1 [file genes-10-00910-s001.zip › Figure S4.pdf]
