# Supplementary material for: Massive Loss of Olfactory Receptors But Not Trace Amine-Associated Receptors in the World’s Deepest-Living Fish (Pseudoliparis swirei)
Source: Genes (Basel). 2019 Nov 8;10(11):910. doi: 10.3390/genes10110910 (PMC6895882; doi:10.3390/genes10110910)
Supplement: Supplementary file 1 [file Figure S1.pdf › Figure S1.pdf]

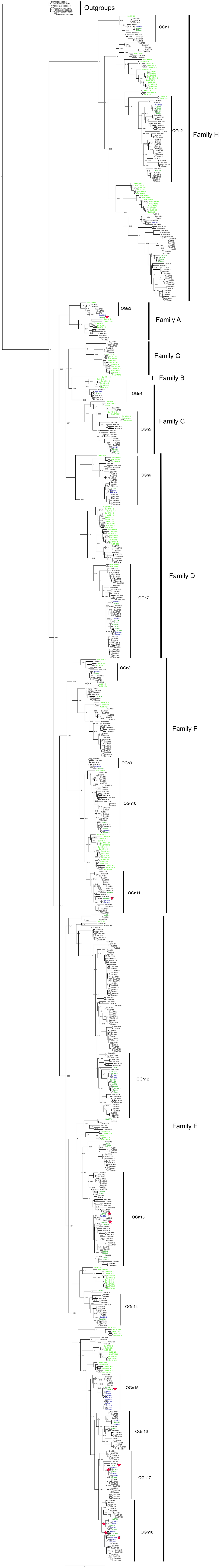
Outgroups
OGn1
OGn2
Family H
OGn3
Family A
Family G
Family B
OGn4
OGn5
Family C
OGn6
OGn7
Family D
OGn8
Family F
OGn9
OGn10
OGn11
OGn12
Family E
OGn13
OGn14
OGn15
OGn16
OGn17
OGn18
